# Development and Comparison of In-house Line Probe Assay (LiPA) and SYBR Green Real-time PCR Regarding the Detection of Periodontal Pathogens

**Published:** 2019

**Authors:** Mohammad Soleimani, Mohammad Reza Zolfaghari

**Affiliations:** Department of Microbiology, Qom Branch, Islamic Azad University, Qom, Iran

**Keywords:** Periodontitis, Real-time PCR, Sensitivity and specificity

## Abstract

**Background:**

Periodontal disease, which can become a chronic condition, is an inflammatory disease that upsets the soft and hard structures supporting the teeth. The aim of the present study was to design and develop an in-house Line Probe Assay (LiPA), to detect putative periodontitis-related bacterial pathogens, and compare it with SYBR Green Real-time PCR.

**Methods:**

The LiPA method was launched using biotinylated 16s rRNA universal primers and specific probes for each of the five bacteria including *Aggregatibacter acti-nomycetemcomitans*, *Prevotella intermedia*, *Tannerella forsythia*, *Porphyromonas gingivalis* and *Treponema denticola*. For this, optimized quantities of the primers and specific probes were dotted onto nylon membrane stripes in a defined pattern. Hybridization was performed between the probes and the single-stranded biotinylated PCR products. The stripes were developed *via* biotin-streptavidin reaction. Ultimately, the analytical and diagnostic sensitivity and specificity of the in-house LiPA was evaluated and compared with SYBR Green Real-time PCR.

**Results:**

The detection limit of the LiPA was 2760 copies of targeted genes. In testing analytical specificity, only signals corresponding to the specific biotinylated products were produced. The calculated diagnostic sensitivity of the LiPA for the five bacterial targets ranged from 96.4 to 100%, whereas the diagnostic specificity was between 90.9 and 100%. Comparing the results, no noticeable difference (p=0.4795) was observed between the two methods.

**Conclusion:**

To screen periodontal pathogens, a simple, inexpensive and accurate method is desirable. The in-house LiPA, having advantages such as high specificity and sensitivity, and the ability to detect five major periodontal pathogens, offers the option of evaluating samples without the need for a post-PCR platform.

## Introduction

As a polymicrobial infection, periodontal disease disturbs tissues supporting and investing the teeth and alveolar bone, and can eventually bring about dental loss. The chronic periodontal diseases are common in the middle-aged and the elderly, while the aggressive periodontal diseases are rampant particularly among youths (30-years-old) 1–3. The prevalence of periodontal diseases in America and developing countries has been estimated about 35 and 50–55%, respectively 4.

During the formation of periodontal disease, the normal oral microbiome shifts gradually from facultative gram-positive bacteria into anaerobic gram-negative ones 1,5,6. *Aggregatibacter actinomycetemcomitans* (Aa) (formerly *Actinobacillus actinomycetemcomitans)* and *Porphyromonas gingivalis* (Pg) are the major pathogenic bacteria species in the etiology of periodontal diseases, whereas the normal oral flora related to the disease are *Prevotella intermedia* (Pi), *Tannerella forsythia* (Tf) and *Treponema denticola* (Td) 7–9.

It is necessary to evaluate microbial composition in human periodontal pockets for the diagnosis and treatment of periodontal diseases 10. Currently, there are various methods to detect the odontopathogen bacteria in saliva or plaque 11. In a former study, conventional PCR and Real-time PCR were developed and compared for detection of Aa and Tf. The SYBR green Real-time PCR was tenfold more sensitive than the conventional PCR 7. Multiplex PCR and Real-time PCR are powerful and efficient tools in detecting concurrently bacterial strains in periodontal samples 12,13. However, multiplex PCR has several drawbacks, including complexity, low amplification efficiency, and poor universality 14. Likewise, the use of Real-time PCR was also complicated with expensive equipment and reagents 15. As a diagnostic molecular method, Line Probe Assay (LiPA) is based on a DNA strip that allows for a rapid and simultaneous identification of the target DNA sequences. In-house and commercially available line probe assays are currently being used to detect the members of the *Mycobacterium tuberculosis* Complex (MTC) and their drug-resistant strains 16–19, and also for genotyping Noroviruses 20, human papillomavirus (HPV) in cervical scrapes 21 and Hepatitis C virus 22–27. The aim of the present study was to design and apply the Line Probe Assay for a rapid screening of several prominent oral pathogens including Aa, Pi, Tf, Pg and Td bacteria.

## Materials and Methods

### Strains and DNA extraction

The genome of wild-type bacteria of Aa (ATCC 700 685D-5), Tf (ATCC 43037D-5), Td (ATCC 35405D-5), Pg (ATCC 33277D-5) and Pi (ATCC 25611D-5) was used as positive control. The bacterial species including *Staphylococcus aureus* (ATCC 25923), *Bacillus subtilis* (ATCC 6051), *Shigella sonnei* (ATCC 92 90), *Escherichia coli* (*E. coli)* (ATCC 25922), *Enterococcus faecalis* (ATCC 29212), *Pseudomonas aeruginosa* (AT CC 27853), *Klebsiella pneumoniae* (ATCC 7881), *Yersinia enterocolitica* (PTCC 1480), *Yersinia pseudotuberculosis* (PTCC 1244), *Proteus vulgaris* (PT CC 10 79) and *Citrobacter freundii* (PTCC 1499) were used as negative control. The bacterial genomes were extracted using EZ-10 spin Column Genomic DNA Kit (DNP™ kit (Bio Basic Inc., Canada)); the purity of the genomes was subsequently evaluated. The extracted genomes were quantified using a Picodrop Spectrophotometer (Picodrop, UK) according to the manufacturer’s instructions.

### Primers and probes

NCBI deposited sequences of *16S rRNA* gene of the target bacteria were downloaded and aligned by CLC Main Workbench software version 7.5 (accession numbers are not shown). A pair of broad range universal bacterial primers was selected to amplify a 466-*bp* region: Forward primer 5′-TCCTACGGGAGGCAGCA GT-3′ and reverse primer 5′-GGACTACCAGGGTAT CTAATCCTGTT-3′^28^.

The 466 *bp* region of the 16S rRNA alignment was designated to design specific probes for each of the five target bacteria through the use of CLC Main Workbench software version 7.5 (Table 1). Confirming the specificity of the designed probes, the sequences were compared against the NCBI nucleotide databases. The biotinylated and non-biotinylated forms of the universal primers and the probes containing 5' poly (T) sequences were commercially synthesized.

**Table 1.** The specific probes for each of the five target bacteria used in the study

**Bacterium**	**Probe name**	**Position**	**Probe sequence (5′-3′)**	**Length (*bp*)**	**Reference**
***A. actinomycetemcomitans***	Agg-p	617–632	CTTTAGGGAGGGGTAG	16	This study
***P. gingivalis***	Por-p	736–751	TTGCCATACTGCGACT	16	This study
***P. intermedia***	Pre-p	662–667	GTGCACGCAACGTATG	16	This study
***T. forsythia***	Tan-p	655–670	TATAGATGAAGTAGGC	16	This study
***T. denticola***	Tre-p	674–689	ATCACGGAGGGGAAAC	16	This study

### PCR and cloning

PCR experiments were conducted in a volume of 25 *μl*, consisting of 1X PCR buffer (Fermentas, GmbH, Germany), 2 *mM* MgCl_2_, 0.2 mM dNTPs, 0.5 *μM* of the non-biotinylated universal bacterial primers, 50 *ng* of genomic DNA (each of the five target bacteria) and 1 unit Taq DNA polymerase (Fermentas, GmbH, Germany). Amplification was carried out for 35 cycles in the PCR program as follows: Initial denaturation at 94*°C* for 4 *min*, denaturation at 94*°C* for 30 *s*, annealing temperature at 54*°C* for 30 *s*, extension time at 72*°C* for 1 *min* and final extension for 10 *min*. The 466 *bp* PCR products of *16S rRNA* genes (each of the five bacteria) were cloned in plasmid vector by use of InsTA clone PCR cloning kit (Fermentas, Lithuania) according to the manufacturer’s instruction. The recombinant plasmids were transformed into CaCl_2_ competent *E. coli* Top10 F′ strain. The confirmed recombinant plasmids containing 16S rDNA insert of Aa, Tf, Td, Pg and Pi were named pTZ-agg, pTZ-tan, pTZ-tre, pTZ-por and pTZ-pre, respectively. The plasmids were subjected to PCR same as above except for the fact that biotinylated primers were used. The biotinylated PCR products were stored at −20*°C* until use in LiPA experiment.

### Preparation of the LiPA strips

The probes (Table 1), the biotinylated universal primers (Conjugate control) and the PCR products from non-biotinylated universal primers (Amplification control), were prepared with concentrations of 2.5 *pmol/μl*; they were then spotted onto SensiBlot™ Plus Nylon membranes (Fermentas GmbH, Germany) employing a 96-well Dot-Slot Blotter (Cleaver, UK) according to the manufacturer protocol. Fixation of the spotted membranes was done through baking at 80°*C* for 30 *min*. Furthermore, the spots were UV cross-linked with 100–150 *μJ/cm*^2^ on the nylon membranes. The prepared membranes were sliced into 60×7 *mm* strips and stored at −20*°C* until use.

### Prehybridization, hybridizations, and color development

The prepared stripes were prehybridized in 2X SSC buffer as blocking solution [1X SSC: 0.15 *M* NaCl (Hi-Media, India) and 0.015 *M* Sodium Citrate (Appli-Chem GmbH, Germany)] and 0.1% SDS (Cinnagen, Iran) at 50 °*C* for 30 *min*. Each prehybridized strip was transferred to a 14 *ml* polystyrene conical bottom test tube containing 2 *ml* hybridization buffer (2X SSC 0.1% SDS). Adding 10 *μl* of the single-stranded biotinylated PCR products, hybridization was accomplished in an orbital shaker at 50*°C* and 80 *rpm* for 1–2 *hr*. Washing the strips twice with 2X SSC and 0.1% SDS for 5 *min* at 22*°C* was followed by washing twice with 1X SSC and 0.1% SDS for 15 *min* at 42°*C*. The hybridized strips were developed via Biotin Chromogenic Detection Kit (Fermentas GmbH, Germany) according to the manufacturer instructions. All steps were performed at 22*°C* with moderate shaking on an orbital platform shaker. Primarily, the strips were treated with 2 *ml* blocking/washing buffer for 5 *min* and then blocked in 2 *ml* of the blocking solution for 30 *min*. Next, the strips were immersed in 2 *ml* of diluted Streptavidin-AP conjugate for 30 *min*. After washing with blocking/washing buffer and incubating with detection buffer for 10 *min*, enzymatic reaction and color development was performed. The strips were incubated in 2 *ml* of fresh substrate solution [5-Bromo, 4-Choloro, 3-Indolylphosphate (BCIP) and Nitro-blue-tetrazolium (NBT)] in a dark place. The blue-purple precipitate was produced after 20–30 *min* of incubation. After discarding the substrate solution, the strips were rinsed with Milli-Q water to stop the enzymatic reaction. Lastly, the strips were interpreted through inspecting the blue-purple bands corresponding to controls and bacterial species.

### Analytical sensitivity and specificity

A 10 *ng/μl* concentration (2.76×10^9^ copies) of the positive control plasmids (pTZ-agg, pTZ-tan, pTZ-tre, pTZ-por and pTZ-pre) was used to prepare a 10-fold serial dilution of each. The dilutions were subjected to PCR amplification using the biotinylated primers. The specific PCR products were detected through the use of the designed in-house LiPA as previously mentioned. The last dilutions demonstrating relevant blue-purple bands on the strips were specified as the lower detection limit of the assay. In order to determine the specificity, four different mixtures of the positive control were prepared as shown in table 2. After that, each mixture was subjected to the designed LiPA according to the above description. For further investigation of the probability of non-target genes detection in other organisms, the genome of negative control bacteria (*Klebsiella pneumonia* ATCC 7881, *E. coli* ATCC 25922, *Bacillus subtilis* ATCC 6051, *Staphylococcus aureus* ATCC 25923, *Enterococcus faecalis* ATCC 29212, *Streptococcus penumoniea* ATCC 700669 and *Neisseria meningitides* ATCC13060) was studied by the designed assay.

**Table 2.** Different mixtures of the positive control prepared in order to determine the specificity

	**pTZ-agg**	**pTZ-por**	**pTZ-pre**	**pTZ-tan**	**pTZ-tre**
**Mixture 1**	10 *ng/μl*	10 *ng/μl*	0	0	0
**Mixture 2**	10 *ng/μl*	10 *ng/μl*	10 *ng/μl*	0	0
**Mixture 3**	10 *ng/μl*	10 *ng/μl*	10 *ng/μl*	10 *ng/μl*	0
**Mixture 4**	10 *ng/μl*	10 *ng/μl*	10 *ng/μl*	10 *ng/μl*	10 *ng/μl*

### Artificially contaminated blood samples

Artificially infected blood samples were used in order to compare the in-house Line Probe Assay and SYBR Green Real-Time PCR concerning the detection of periodontal pathogens. For this purpose, a normal human EDTA-treated whole blood sample was utilized. The sample was distributed into thirty micro tubes (200 *μl/tube*) which were subsequently spiked with the genomic DNA of the five periodontal pathogens as follows: The tubes were inoculated with 1 *μg*/200 *μl* final concentration of the genomic DNA of each pathogen. Moreover, ten blood samples (not spiked with DNA) were used as negative control. The artificially contaminated samples and the negative control samples were kept at 4°*C* for 96 *hr*. Then, forty samples were subjected to DNA extraction using EZ-10 spin Column Genomic DNA Kit (DNP™ kit (Bio Basic Inc., Canada). The DNA samples were examined by the in-house Line Probe Assay as mentioned above. Also, SYBR Green Real-time PCR was employed to detect the pathogens in the samples. ABI 7500 Fast Real-Time Amplification system (ABI, USA) and SY-BR® Premix Ex Taq™ II (TAKARA, Japan) were utilized in all experiments. Each extracted DNA and the negative control were subjected to the SYBR Green Real Time PCR to detect each of the five periodontal pathogens, separately. Additionally, the purified genomic DNAs were used as positive control. The reaction tubes were prepared in a final volume of 20 *μl*, containing 2 *μl* of extracted genomic DNA as a template, 2 *μl* of 2X master mix and 0.4 *M* of each of the forward and reverse primers (Table 3). The thermal cycling program was a 10 *min* activation of Taq polymerase at 95°*C* by 40 cycles of PCR (denaturation at 95*°C* for 15 *s*, annealing and extension at 60*°C* for 1 *min*). Finally, to verify the correct product by its specific melting temperature (Tm), a melting curve analysis of the amplified DNA was performed at 54 to 95*°C* and a temperature increase rate of 0.2*°C*/sec was considered in this test.

**Table 3.** Characteristics of the primer pairs utilized in the study

**Bacterium**	**Target gene**	**Primer name**	**Position**	**Primer sequence**	**Reference**
***A. actinomycetemcomitans***	*hbpA*	*F-Aggrega-hbpA*	2614 – 2634	AGACCCAATGCAAAAGTAACG	(7)
		*R-Aggrega-hbpA*	2774 – 2756	GCAGTTCTGGGCTGAATTG	
***P. gingivalis***	*fimA*	*F-Porphy-fimA*	296 – 313	ACAGCAGGAAGCCATCAA	Unpublished data
		*R-Porphy-fimA*	457 – 438	GCAGTCAGTTCAGTTGTCAA	
***P. intermedia***	*16S rRNA*	*F-Pre 16*	357–375	GAGGCAGCAGTGAGGAATA	Unpublished data
		*R-Pre 16*	637–618	GCAAGGTAGATGTTGAGCAC	
***T. forsythia***	*16S rRNA*	*F-Tan-16*	469–490	GCATGTACCTTGTGAATAAGCA	(7)
		*R-Tan-16*	718–700	CTTCGCAATCGGAGTTCTG	
***T. denticola***	*16S rRNA*	*F-Tre-16*	266 – 284	CAAGGCAACGATGGGTATC	Unpublished data
		*R-Tre-16*	438 – 414	CTGCAAAAGAATTTTACAACCTTTC	

### Statistical analysis

The online Clinical Calculator 1 was used to calculate the clinical sensitivity and specificity of the LiPA and the SYBR Green Real-time PCR (http://vassars-tats.net/clin1.html). Clinical sensitivities of the assays were compared by McNemar’s test using an online calculator (https://graphpad.com/quickcalcs/mcNemar1/).

## Results

### LiPA setting up

**Figure 1.**
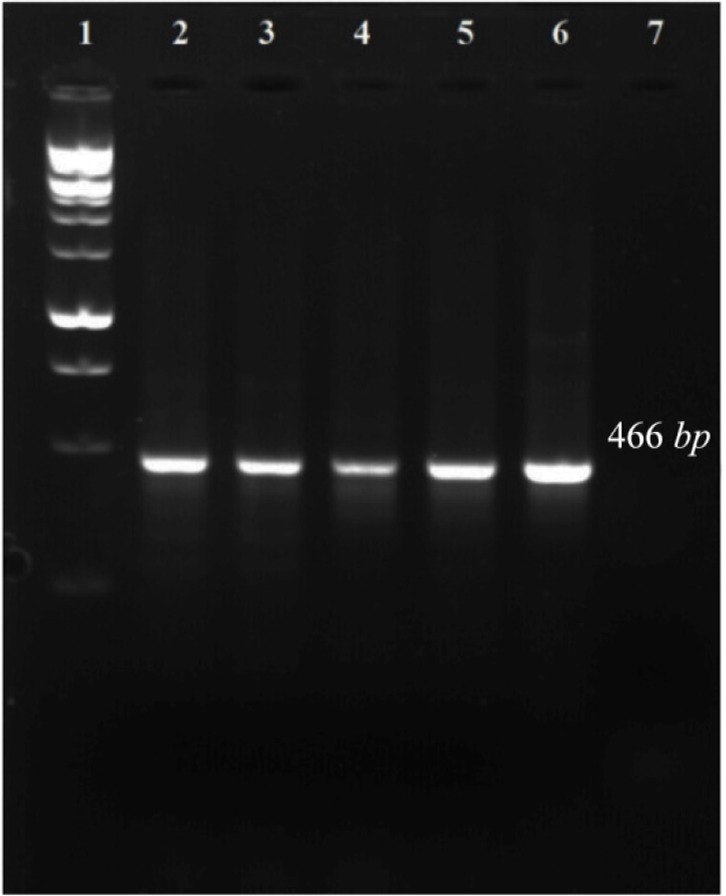
PCR amplification of 16s rRNA gene related to five target bacteria. 1: 100 *bp* DNA ladder, 2–6: 7: negative control.

**Figure 2.**
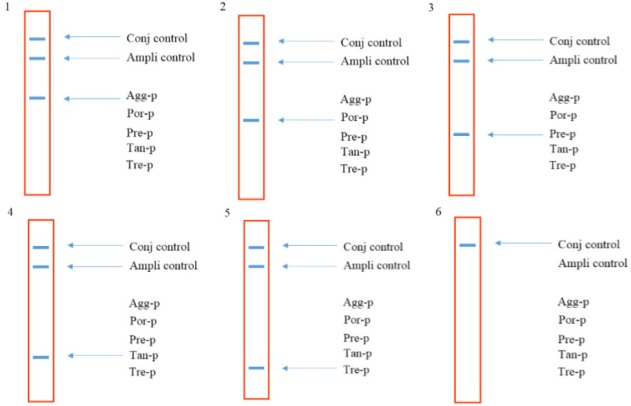
The schematic strips demonstrating the presence of the bacterial species based on the reaction between specific probes and corresponding biotinylated PCR product. 1: Aa, 2: Pg 3: Pi, 4: Tf, 5: Td and 6: negative control (without DNA).

A 466 *bp* region from the *16s rRNA* gene of the five target bacteria was amplified and visualized on gel agarose. The PCR product lengths were the same regardless of the bacterial species (Figure 1). In preparing the positive control plasmids, the 466 *bp* PCR products were separately cloned in pTZ57R/T plasmid. The PCR and sequencing of the inserts confirmed the recombinant plasmids (pTZ-agg, pTZ-tan, pTZ-tre, pTZ-por and pTZ-pre). In the setup of the in-house LiPA, each target gene was minutely detected. In all strips, one universal probe was used to detect bacterial *16S rRNA* gene (Ampli control) and a conjugate control (Conj control) to confirm biotin and streptavidin reaction. In all runs, bands corresponding to Ampli control and Conj control showed blue-purple precipitate; however, in negative control cases (without DNA) only the band of Conj control was visualized. In addition, in each experiment, only the blue-purple precipitate related to the applied specific biotinylated product was produced (Figure 2).

### Analytical sensitivity and specificity results

**Figure 3.**
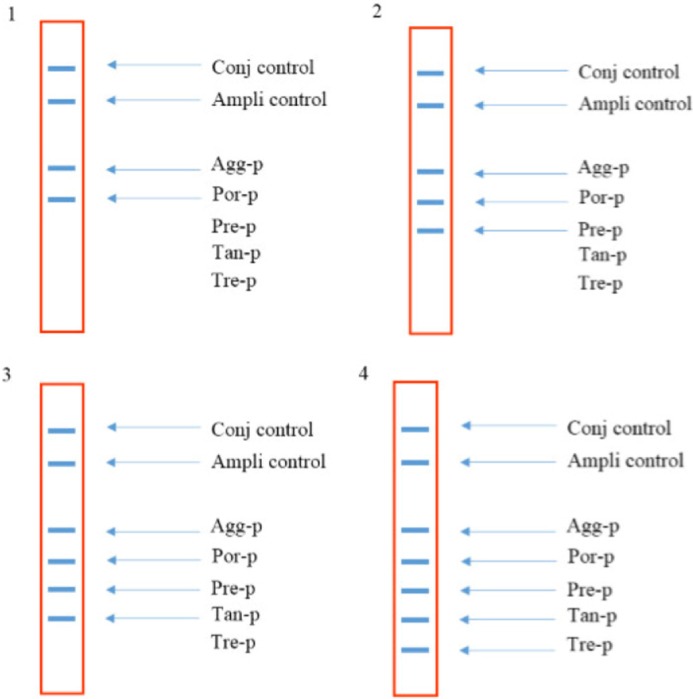
The schematic strips demonstrating the specificity of the in-house LiPA. 1) signals produced when using pTZ-agg and pTZ-por plasmids, 2) signals produced when using pTZ-agg, pTZ-por and pTZ-pre plasmids, 3) signals produced when using pTZ-agg, pTZ-por, pTZ-pre and pTZ-tan plasmids and 4) signals produced when using pTZ-agg, pTZ-por, pTZ-pre, pTZ-tan and pTZ-tre plasmids.

Sensitivity results showed that the last dilution of the positive control plasmids (pTZ-agg, pTZ-tan, pTZ-tre, pTZ-por and pTZ-pre), generating a blue-purple precipitate on the strips, was a 10 *fg* dilution. Accordingly, the LOD of the test was determined 10 *fg* or 2760 copies of each of the five target genes. To determine the specificity, the in-house LiPA was performed on four different mixtures of the positive control plasmids. In each experiment, only signals corresponding to the specific biotinylated products were produced (Figure 3). Using the negative control bacterial genomes in the in-house LiPA, only bands pertaining to Ampli control and Conj control indicated the blue-purple precipitate on the strip.

### Results of artificially contaminated blood samples

**Figure 4.**
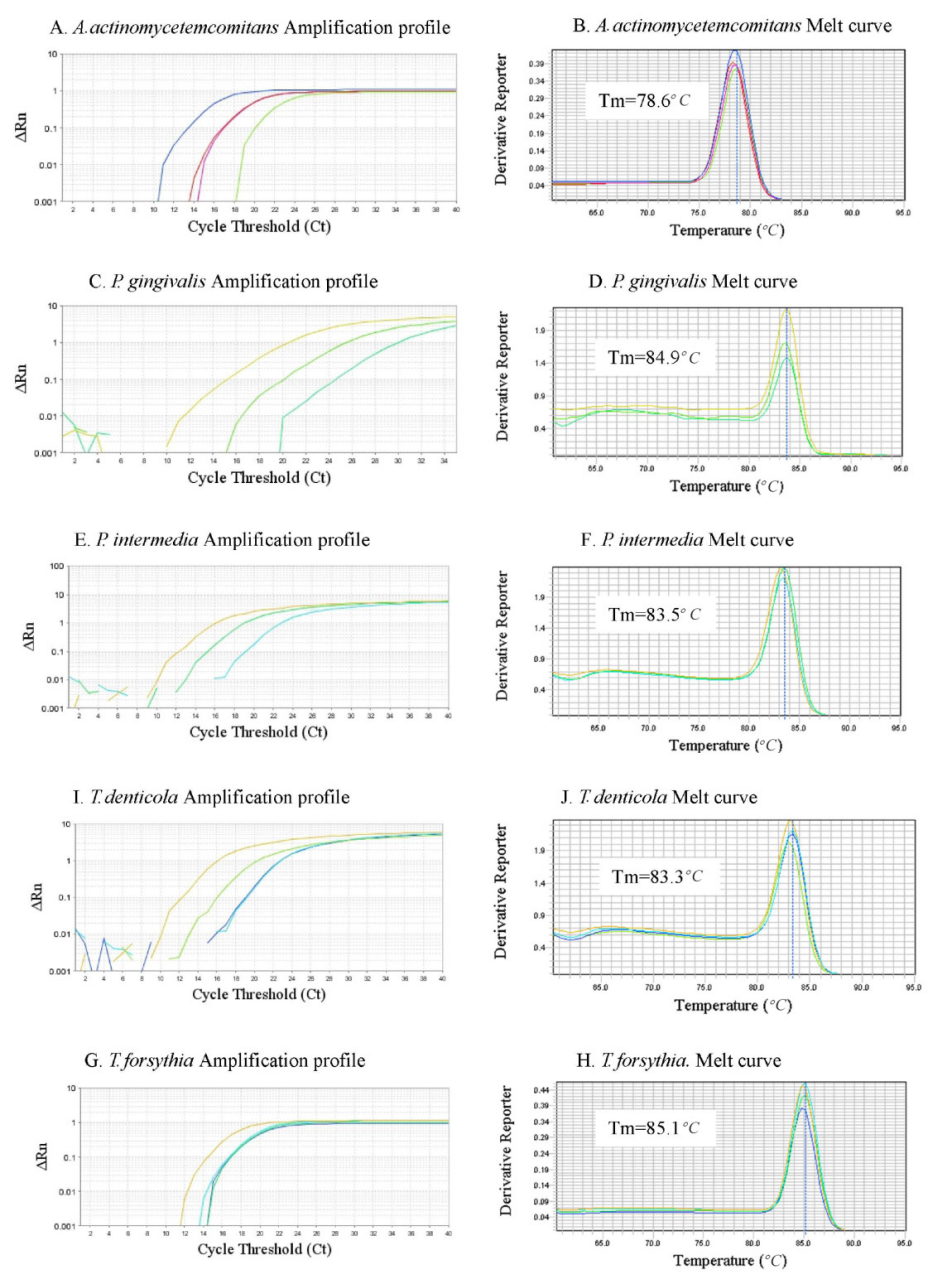
The amplification profiles and the corresponding melt curves of the periodontal pathogen, the melting temperature (*T*m) of each amplicon is shown along with its melt curve.

The results of performing the LiPA on the DNA extracted from the spiked and non-spiked blood samples were inspected based on the relevant bands on the strips. In SYBR Green Real-time-PCR, a sample is considered positive when the amplification plot crosses the threshold value. The amplification plot of Aa contaminated samples exceeded the threshold baseline at a Ct value of 10–18, whereas the amplification plot of the negative control samples did not exceed the threshold baseline. Figures 4A, C, E, G, I show the Ct values of amplification plot related to the five periodontal pathogens. To ensure that the amplification plot obtained for the artificially contaminated sample was precise enough, Tm analyses of the assays were performed (Figures 4B, D, F, H, J). Table 4 illustrates the diagnostic sensitivity and specificity of the in-house LiPA and McNemar p-value when the SYBR Green Real-time PCR was used as gold standard. All the negative samples in the periodontal pathogen DNA (non-spiked samples) of the LiPA were also negative in SYBR Green Real-time PCR assay. Comparison of the results indicated no significant difference between the two methods (p-value=0.4795 for Aa, Pi and Td and 1 for Pg and Tf).

**Table 4.** Comparison of SYBR Green Real-time PCR and in-house LiPA concerning the detection of DNA extracted from artificially contaminated blood samples

**SYBR Green Real− time PCR+**		**SYBR Green Real time PCR−**	**Total**	**Diagnostic sensitivity**	**Diagnostic specificity**	**McNemar p-value**
***A. actinomycetemcomitans***						
LiPA+	27(67.5)	1(2.5)	28	96.4%	91.6 %	0.4795
LiPA−	1(2.5)	11(27.5)	12			
Total	28	12	40			
***P. intermedia***						
LiPA+	27(67.5)	1(2.5)	28	96.4%	91.6 %	0.4795
LiPA−	1(2.5)	11(27.5)	12			
Total	28	12	40			
***T. denticola***						
LiPA+	28(70)	1(2.5)	29	96.5%	90.9 %	0.4795
LiPA−	1(2.5)	10(25)	11			
Total	29	11	40			
***P. gingivalis***						
LiPA+	29(72.5)	0	29	96.6%	100 %	1
LiPA−	1(2.5)	10(25)	11			
Total	30	10	40			
***T. forsythia***						
LiPA+	29(72.5)	0	29	100%	100 %	1
LiPA−	0	11(27.5)	11			
Total	29	11	30			

## Discussion

In this study, 16S rRNA-based in-house LiPA was designed and applied so as to detect five major putative periodontal pathogens. 16S rRNA is an important housekeeping gene that shares a high degree of similarity in different bacterial species. Using CLC Main Workbench software, the unique regions of the gene in each target bacterium were found and used as species-specific probe. Culture-based methods are technique-sensitive, time-consuming and expensive. In addition, they require a great amount of expertise and are difficult to perform, rendering such methods inappropriate for routine bacterial screening in the periodontal identification 29. Nowadays, the PCR method has become a powerful tool for the identification of various organisms, particularly those showing problems with regard to phenotypic methods.

Bacterial *16S rRNA* genes contain the interspersion of more- and less-conserved sequences; hence their application as a diagnostic gene in molecular detection is reasonable. The more distinct regions can be used to discriminate the bacterial taxa, while the conserved regions present universal sequences in annealing the PCR primers 30. Several studies have demonstrated the usefulness of 16S rRNA-based PCR detection of periodontal bacteria in subgingival samples. 16S rRNA PCR methods are more sensitive than culture-based approaches and less cross-reactive compared with DNA probe detection 7,11,31,32.

In this study, a pair of broad range universal bacterial primers was used 28 to amplify the *16s rRNA* gene of five target bacteria. The sequence alignment was used to identify a specific region of each *16S rRNA* gene, hence a candidate for specific probe. The preparation, hybridization, and development of the strips were subsequently performed.

In a previous study, to genotype Hepatitis C Virus (HCV) types and subtypes, the suitability of the work-flow was shown, particularly the use of Biotin Chromogenic detection kit for the development of the strips 33. This is the first time that some of the most important periodontal pathogens are evaluated by an in-house line probe assay. With the in-house LiPA, the extracted DNA of artificially contaminated blood samples was evaluated and the results were compared with the SYBR Green Real-time PCR. It was concluded that there is concordance between results of the LiPA and that of SYBR Green Real-time PCR as far as detecting the five periodontal pathogens is concerned. Aberle *et al* compared LiPA detecting drug-resistant Hepatitis B Virus (HBV) strains with sequence analysis 34. They obtained results concordant with sequence analysis with 48 of 56 serum samples from HBV-infected patients undergoing lamivudine therapy. Several studies have proposed LiPA as a screening test with low turnaround time, especially in low-resource regions 35,36. For example, Albert *et al* showed its usefulness for rapid screening of MDR-TB in Uganda 35. Melchers *et al* applied LiPA to detect and genotype 16 different Human Papillomaviruses (HPV) types simultaneously, where it was shown that the method was sensitive, specific, simple, and fast. Also, they proposed HPV-LiPA to mass screen the cervical samples for routine analyses and patient management 37.

## Conclusion

To screen periodontal pathogens, a simple, inexpensive and accurate method is required, so a reliable, sensitive, specific and economic detection assay was developed and applied in this study. LiPA provides the possibility of evaluating more species without the need for gel agarose electrophoresis step and can, therefore, be a useful candidate for research and diagnostic purposes of oral microbiology.
